# Supplementary material for: D2I and F9Y Mutations in the NS1 Protein of Influenza A Virus Affect Viral Replication via Regulating Host Innate Immune Responses
Source: Viruses. 2022 Jun 1;14(6):1206. doi: 10.3390/v14061206 (PMC9228823; doi:10.3390/v14061206)
Supplement: Supplementary file 1 [file viruses-14-01206-s001.zip › viruses-1712771-supplementary.pdf]

viruses

Table S1. Identified NS1 mutations of 24 purified plaques from passaged NS1 random mutant library virus.

Virus Number	NS1(nt) mutation	NS1 aa mutation
1	*a172t+a435g*	T58S+I145M
2	- [a]	-
3	-	-
4	*a661g*	K221E
5	*c245t*	A82V
6	*t284c*	L95P
7	*t26a*	F9Y
8	-	-
9	-	-
10	*t26a*	F9Y
11	*a11c+t284a+ a515c*	N4T+L95H+E172A
12	g4a a5t+t26a	D2I+F9Y
13	g4a a5t+a172t	D2I+T58S
14	-	-
15	-	-
16	*t26a*	F9Y
17	*a172t+a435g*	T58S+I145M
18	-	-
19	-	-
20	*t26a*	F9Y
21	-	-
22	-	-
23	*t26a*	F9Y
24	-	-

[a] unmutated.
